# Are we really “eating well with Canada’s food guide”?

**DOI:** 10.1186/s12889-018-5540-4

**Published:** 2018-05-22

**Authors:** Joyce J. Slater, Adriana N. Mudryj

**Affiliations:** Human Nutritional Sciences, University of Manitoba, 409 Human Ecology Building, Winnipeg, MB R3T 2N2 Canada

## Abstract

**Background:**

Canada’s Food Guide (CFG) has been an important health promotion tool for over seventy years. The most recent version was released in 2007. This study examined Canadians’ exposure to, knowledge, and use of CFG.

**Methods:**

Data came from the Canadian Community Health Survey’s Rapid Response on the Awareness and Usage of Canada’s Food Guide, which included 10,098 Canadians ≥12 y in all ten provinces. Questions were asked on familiarity, awareness and usage of CFG and Canada’s Food Guide for First Nations, Inuit and Métis, as well as healthy eating principles and behaviours. Descriptive statistics and logistic regression were used to observe counts and differences among key demographic variables.

**Results:**

More than 80% of Canadians have heard of CFG however significantly more women than men were aware of the Guide. Most knew that ‘Vegetables and Fruit’ had the most recommended servings and that dark green vegetables should be consumed daily; however fewer than half knew this of orange vegetables. Just under one third had a copy in their homes, and the most common sources for obtaining CFG were child’s school and health professional/trainer. Those who consulted CFG recently were more likely to consume the recommended servings of vegetables and fruits, and to state that their eating habits were ‘much better’ than one year previously.

**Conclusions:**

CFG has “brand recognition” among Canadians however there are gaps between awareness and eating behaviours. The new Food Guide could consider additional dissemination tools including social media, videos and workbooks tailored to various age groups, demographic groups and settings.

## Background

While humans have had guidelines for what to eat for millennia, many of these were religious in nature, and may or may not have been rooted in public health. Dietary guidelines for health and disease prevention, as put forth by governments and public health agencies are a relatively modern phenomenon. Their original purpose was to deal with food shortages resulting from war, natural disasters or distribution problems [1]. The discovery of vitamins prompted a new wave of “dietary standards” to improve national diets. In Canada, the first dietary standards were developed in 1938 by the inaugural Canadian Council on Nutrition. One of the imperatives of the standards was to provide scientific evidence for dietary quality and quantity, in support of increasing unemployment relief benefits during the Great Depression [1].

World War II saw the return of food rationing which spurred the development of Canada’s first food guidelines in 1942 [2]. The aim of “Canada’s Official Food Rules” was to prevent nutrition deficiencies while coping with rationing, and introduced the concept of food groups, emphasizing the consumption of nutrient-rich milk, especially by children. Two more versions of “Food Rules” followed in 1944 and 1949, followed by the first version of “Canada’s Food Guide” in 1961. In addition to recommendations based on five food groups and serving sizes for different ages categories, the Guide offered information on vitamin D, shopping, feeding infants and meal planning [2].

The three subsequent versions of “Canada’s Food Guide” (1977, 1982, 1992) featured the now-familiar four food groups, with more inclusive ‘Meat and Alternatives’ and ‘Milk and Alternatives’, and combined the deficiency prevention messages of previous guildelines with those focused on chronic disease prevention. These included “moderation” and “energy balance” in recognition of changing disease patterns in the population [2]. The latest 2007 version is a booklet titled “Eating Well with Canada’s Food Guide”, and continues this trend with additional guidance on the ‘Vegetables and Fruit’ group with nutrition promotion messages for different ages and life stages [3]. Further, this version of the Food Guide was produced in multiple languages to reflect changing population demographics. It was also adapted for Canadian Indigenous groups as “Eating Well with Canada’s Food Guide: First Nations, Inuit and Métis” [4].

Canada’s Food Guide, far from being a benign health promotion tool, has long sparked controversy for including industry representatives in its development, as well as being criticized for potentially contributing to, rather than preventing, obesity [5–7]. Nonetheless, according to various media sources [8] Canada’s Food Guide is the most sought Government document after income tax forms. However, despite its long-standing presence in Canadian society, and it’s apparent popularity, there has not been much research into its usefulness to Canadians, or effectiveness in modifying their diets. That which has been conducted has mainly been on small to modest numbers of participants [9–11]. Assessing the impact of Canada’s Food Guide at a population-level is warranted to inform nutrition programs and policies which are based on the Food Guide and its principles. Therefore the purpose of this study was to examine Canadians’ exposure to, and use of, Canada’s Food Guide, and its role in healthy eating using data from the 2012 Canadian Community Health Survey. This research is doubly important right now, as the Canadian Government recently announced plans to update Canada’s Food Guide [12]. Understanding Canadian’s perceptions and usage of the 2007 Guide will be useful for educators and policy-makers, as well as provide critical information for the development of the next version.

## Methods

This research is based on results from the Canadian Community Health Survey’s (CCHS) Rapid Response on the Awareness and Usage of Canada’s Food Guide, which collected data from a nationally representative sample of Canadians ≥12 y in all ten provinces. Data collection took place across the country in May and June of 2012. Trained interviewers from Statistics Canada administered questions related to eating habits and knowledge of Canada’s Food Guide and other healthy eating principles, including whether or not the respondent consulted any sources to learn more about healthy eating (i.e. health professionals, food companies, family or friends or Canada’s Food Guide). Interviews were conducted in person for each respondent, or by proxy if the mental or physical health of the respondent made it impossible to conduct the interview. In total, 10,098 respondents completed the module [13].

This study focused on the self-rated familiarity, awareness and usage of Canada’s Food Guide (and Canada’s Food Guide for First Nations, Inuit and Métis), including questions about general awareness of the Food Guide (*Have you ever seen or heard of Canada’s Food Guide? Have you ever looked through it?*) as well as reasons for looking through it or not looking through it and whether or not the respondents had a copy of the Food Guide at home, and where they obtained it from. Other questions pertained to whether or not the respondent used information from the Food Guide to influence their diet, and inquired about their eating habits and food group consumption knowledge. Respondent’s knowledge of food group consumption and healthy eating principles were assessed by asking which food group should be consumed most (serving wise) per day, as well as by asking questions about quantifying consumption of key foods (dark leafy greens and orange coloured vegetables) on a scale ranging from once a day to twice a month [14].

Demographic variables such as gender, age, income, education and marital status were used to assess the variability of these practices across Canada, and key variables were examined to assess differentiations between various socio-demographic groups. Descriptive statistics were used to observe differences among gender, in addition to Aboriginal status, immigrant status, and comparisons between family structure and other characteristics were observed by looking at marital status and respondent and household education. Income was also examined as a factor, splitting respondents into 4 groups based on their household income. Logistic regression was used to determine whether any of the aforementioned demographic variables were associated with food guide knowledge or use. Odds ratios were calculated and the significance level was set at *P* < 0.05.

All analyses were performed using PASW SPSS Statistics [15] and STATA Statistical Software [16]. Because the CCHS uses a multi-stage stratified complex survey design and requires a complex formula to calculate variance estimates, Statistics Canada recommends using bootstrapping to estimate distribution from a sample’s statistics. The bootstrapping method was used in all the data analyses for the present study via STATA software [16]. Research for this study was conducted at the Manitoba Research Data Centre and was consistent with Research Ethics Board Requirements. Project approval was granted by Statistics Canada, allowing project members to access the data. Data was analyzed in a secure environment and all output was vetted prior to its release.

## Results

**Fig. 1:**
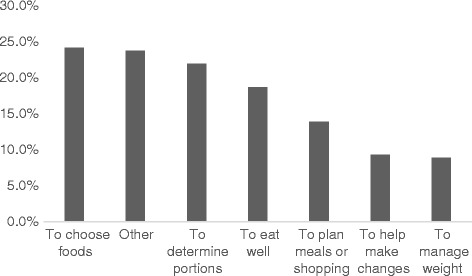
Reasons for using Canada’s Food Guide/Canada’s Food Guide for First Nations, Inuit and Métis (respondent marked all that applied)

**Fig. 2:**
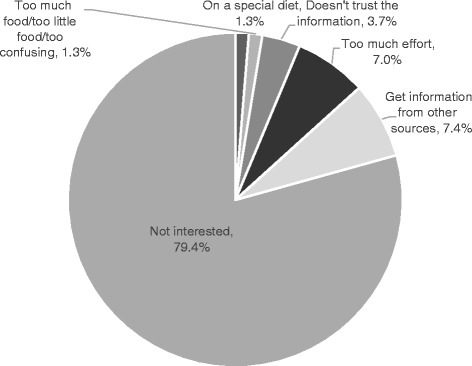
Main reasons for not looking at Canada’s Food Guide/Canada’s Food Guide for First Nations, Inuit and Metis (Respondent marked all that applied)

Canadians had a high level of awareness of Canada’s Food Guide. More than four out of five had heard of the Food Guide, with significantly more women being aware than men (Table 1). Of those aware of the Food Guide and utilizing it, the main reasons were “to choose foods”, “determine portions” and “to eat well” (Fig. 1). Of those who had heard of the Food Guide, 15.9% of women had never looked through the Guide, while one third (33.6%) of men had never looked through it (Table 1). Reasons for not looking through it are shown in Fig. 2 with the vast majority expressing no interest in it.

**Table 1:** Knowledge and awareness of Canada’s Food Guide

Have you heard of Canada’s Food Guide to healthy eating?		
	% Yes	Odds ratio (CI)
Male	80.6	0.64*** (0.53–0.78)
Female^a^	86.6	1.00
Overall	83.6	
Have you ever looked through it (if yes to previous question)		
	% Yes	Odds ratio (CI)
Male	66.4	0.37*** (0.31–0.45)
Female^a^	84.1	1.00
Overall	75.5	
Have you ever heard of Canada’s Food Guide for first nations, inuit or métis?^b^		
	% Yes	Odds ratio (CI)
Male	28.6	1.35 (0.70–2.61)
Female^a^	22.9	1.00
Overall	25.9	
Have you ever looked through it (if yes to previous)		
	% Yes	Odds ratio (CI)
Male	62.1	0.93 (0.32–2.66)
Female^a^	63.9	1.00
Overall	62.8	
Do you have a paper copy of Canada’s Food Guide^c^ at home?		
	% Yes	Odds ratio (CI)
Male	21.9	0.42*** (0.36–0.50)
Female^a^	39.8	1.00
Overall	31.4	
Have you ever accessed Canada’s Food Guide^c^ on the internet?		
	% Yes	Odds ratio (CI)
Male	27.1	0.82*** (0.70–0.95)
Female^a^	31.3	1.00
Overall	29.4	

^a^Reference group

^b^If respondent identified self as First Nations, Inuit or Métis

^c^Canada’s Food Guide to Healthy Eating, or Canada’s Food Guide for Fist Nations, Inuit or Métis

*p < 0.05, **p < 0.01, ***p < 0.001

**Fig. 3:**
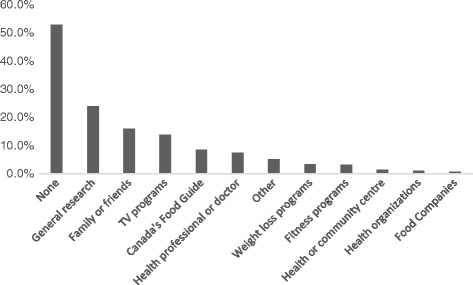
Sources of healthy eating information consulted in the last six months (respondent marked all that applied)

Respondents identified consulting a variety of sources to learn about healthy eating (Fig. 3). The most frequent answer was no sources (53%), followed by general research (24%) and family and friends (16%). Only 8.7% stated having consulted the Food Guide within the last six months.

**Fig. 4:**
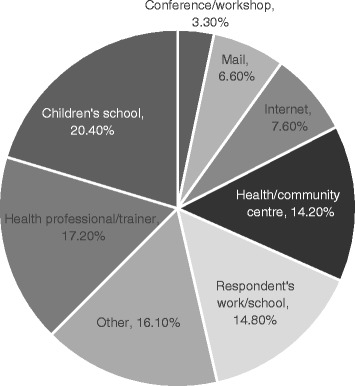
Where most recent copy of Canada’s Food Guide/Canada’s Food Guide for First Nations, Inuit and Métis was obtained

Just under one third of respondents had a copy in their home, however significantly more women than men had copies. One fifth of Canadians who had a Food Guide obtained it from their children’s school (Fig. 4). A health professional/trainer was the next most commonly cited source of respondents’ most recent copy of the Food Guide, followed closely by work/school and a health or community centre. Only 7.6% obtained their copy from the Internet.

For Indigenous respondents, just over one quarter of males, and slightly fewer than one quarter of females had heard of Canada’s Food Guide for First Nations, Inuit or Métis (CFGFNIM). Of those who had heard of CFGFNIM, just over one third had never looked through it.

Canadians’ knowledge of the nutrition content of Canada’s Food Guide varied (Table 2). The majority correctly reported that ‘Vegetables and Fruit’ was the food group we should eat the most servings of; however one in ten men thought the most servings should come from ‘Meat and Alternatives’ while one in ten thought it was ‘Grain Products’. The majority also correctly reported that we should eat dark green vegetables every day though less than half correctly reported the same for orange coloured vegetables. More than two-third of Canadians reported that their eating habits were about the same or worse than one year previously (Table 3) and only 12.5% stated they were ‘much better’. Of those who had consulted Canada’s Food Guide in the past six months, almost one quarter stated they had ‘much better’ eating habits. This was more than triple those who did not consult any sources, and higher than those who consulted sources other than Canada’s Food Guide.

**Table 2:** Food Guide nutrition knowledge of Canadians

What is the food group we should eat the most servings of?				
	Grain Products	Milk and Alternatives	Vegetables and Fruit	Meat and Alternatives
	%	%	%	%
Male	9.6	5.2	74.8	10.4
Female	6.7	5.2	83.1	5.0
Overall	8.1	5.2	79.1	7.6
	How often should you eat dark green vegetables?		How often should you eat orange coloured vegetables?	
Once a day	%		%	
Male	74.9		43.6	
Female	77.4		47.0	
Overall	76.2		45.3	
3 times per week				
Male	18.3		43.5	
Female	20.6		42.9	
Overall	19.4		43.2	
Once a week				
Male	3.8		11.0	
Female	4.0		8.8	
Overall	3.9		9.9	
Twice a month				
Male	0.6		1.9	
Female	0.3		1.3	
Overall	0.4		1.6

**Table 3:** Self-assessed eating habits of Canadians (compared to one year ago)

Compared to one year ago, how are your eating habits now?				
	Much better	Somewhat better	About the same	Somewhat or much worse
	%	%	%	%
Male	11.2	20.1	64.6	4.1
Female	13.8	19.4	60.7	6.1
Overall	12.5	19.8	62.6	5.1
If respondent consulted Canada’s Food Guide in the past 6 months to learn more about healthy eating:				
Overall	23.8	27.1	45.9	3.2
If respondent consulted any other source in the past 6 months besides Canada’s Food Guide^a^ to learn about healthy eating:				
Overall	18.8	25.5	51.4	4.3
If respondent did not consult any source in the past 6 months to learn about healthy eating:				
Overall	7.1	14.8	72.4	5.8

^a^Including health professionals such as a family doctor or dietitian, food companies, health organizations, TV programs or weight loss programs

Canadians who consulted Canada’s Food Guide within the past six months were significantly less likely to eat in restaurants or take out meals (Table 4). They were significantly more likely to have ten or more servings, and significantly less likely to have fewer than five servings, of fruits and vegetables. There was no significant difference in BMI category based on Food Guide consultation.

**Table 4:** Dining out/Body Mass Index/fruit and vegetable consumption of Canadians

	Respondents who consulted Canada’s food guide in the past 6 months		Respondents who did not consult any source to learn about healthy eating
	%	Odds ratio (CI)	%
Eating out/Ordering out from restaurants/cafeterias			
0–1 time per week^a^	80.8	1.00	73.8
> 1 time per week	19.2	0.67** (0.50–0.89)	26.2
BMI class^b^			
Normal weight^a^	44.8	1.00	46.6
Overweight	31.7	0.93 (0.69–1.27)	35.1
Obese	23.5	1.34 (0.98–1.82)	18.2
Fruit and vegetable consumption^c^			
< 5 servings/day	42.0	0.43*** (0.34–0.55)	64.0
5–10 servings/day^a^	49.7	1.00	32.5
> 10 servings/day	8.3	1.52*** (0.21–0.31)	3.5

^a^Reference group

^b^According to World Health Organization (WHO) classifications and based on self-reported height and weight

^c^Self-reported

*p < 0.05, ** *p* < 0.01, *** *p* < 0.001

## Discussion

Canadians’ awareness, perceptions and use of Canada’s Food Guide were described in this study, along with their self-reported eating habits. Overall, results indicate that while most Canadians are aware of the Food Guide, and most have basic knowledge of food groups, serving proportions and the importance of fruits and vegetables, far fewer actually use it for healthy eating guidance. This is consistent with other smaller studies which have shown high levels of awareness of Canada’s Food Guide, but low levels of adherence [9–11, 17].

These observations are not surprising for two reasons. First, the healthy eating messages of Canada’s Food Guide exist amidst a veritable sea of nutrition and “healthy eating” information originating from government, non-governmental and private sector organization. Indeed, Canada’s Food Guide was the 6th cited source of healthy eating information by participants. In Canada the weight loss industry is valued at $7 billion per year [18] and a significant portion is devoted to “nutrition” in the form of diet books, web-sites, special food products (with health claims) and supplements. All of these compete for consumers’ attention when they are actively or passively seeking “healthy eating” information, and for-profit sources have significantly more resources to reach consumers. For example, in just over two months in 2016, marketers of weight loss products and services spent over $100,000,000 on television advertising alone [19]. This dwarfs the resources spent promoting healthy eating in Canada. In the 2015–16 fiscal year, a mere $4,571,677 was allocated to federal “nutrition policy and promotion” programming [20].

Second, Canadians are consuming a high proportion of foods that do not fit into the traditional “four food groups” primarily due to their “ultra-processed” composition. Ultra-processed foods are defined as those “made from processed substances extracted or refined from whole foods… Most are made, advertised, and sold by large or transnational corporations and are very durable, palatable, and ready to consume, which is an enormous commercial advantage over fresh and perishable whole or minimally processed foods … [They] are typically energy dense; have a high glycaemic load; are low in dietary fibre, micronutrients, and phytochemicals; and are high in unhealthy types of dietary fat, free sugars, and sodium” [21, 22].

The 2004 Canadian Community Health Survey (Nutrition) found that over ¼ of Canadians’ calories were coming from foods outside the four food groups [23]. However more recent data suggests that 62% of Canadians calories come from ultra-processed foods [22]. These foods are embedded in the way we now eat, such as outside the home and outside traditional meal structures, favouring convenient “on the go” eating which includes few whole foods as depicted in the Food Guide. Consequently, the Guide appears to be “out of step” with Canadian dietary patterns, and may be regarded as irrelevant by consumers choosing less traditional food preparation and eating styles, and embracing a more ultra-processed diet.

Nonetheless, results did show that the Food Guide may confer some protective dietary factors. More Canadians who consulted the Guide with in the last six months felt their eating habits were “much better” than those who did not. They also tended to eat out less, and ate more fruits and vegetables. This does not indicate causation, as those more inclined to eat fruits and vegetables may seek out health promotion resources that reinforce their health behaviour practices. It does, however, suggest that the Food Guide may be an important tool for a segment of the population. This highlights how it is critically important that the food guide be evidence-based as it does appear to influence dietary behaviour of a considerable segment of the population, and therefore may impact long-term health. Further, consumers may be exposed to Food Guide messaging even if they have not seen an official copy of the Guide. Food manufacturers often promote their products through front of package messages (i.e. “contains 1 serving of whole grains”). This extended reach of the Guide through marketing further emphasizes the need for future Food Guides to be evidence-based.

Health care settings, workplaces and schools were predominantly where Canadians are accessing the Food Guide, and should continue to be utilized as venues through which to amplify the Guide’s healthy eating messages. However as discussed, simply handing out the Guide is failing to translate into healthy eating patterns. The current Guide has few contextual teaching tools associated with it, such as lesson plans, videos, or self-guided workbooks that could be tailored to various age groups, demographic groups, and settings such as schools and health care facilities [24]. Further, any such tools would likely have greater impact if used by educators who have received appropriate training. The low rate of Indigenous respondents having heard of Canada’s Food Guide for First Nations, Inuit or Métis suggests more education and resources should be directed here. However, the sample of Aboriginal Canadians in the Canadian Community Health Survey excludes those living on reserve, so the magnitude of awareness in this population is unknown.

Despite the study’s strengths, including its large sample size, it also has limitations. The data are all self-reported and may have created social desirability bias for positive behaviours such as reporting on fruit and vegetable consumption, and improvement of eating habits. Further research should investigate sub-populations’ perceptions of the Food Guide such as different age groups and newcomers, as well as why so many Canadians report being “not interested” in the Guide.

## Conclusions

Canada’s Food Guide is an important health promotion tool, one that has tremendous “brand recognition” among Canadians which is a great asset going forward into the development of the new Food Guide. This is an excellent opportunity to not only construct a new Guide that considers Canadians’ contemporary eating habits, the food environment, and shifting demographics including the influx of newcomers to Canada, but to also strategize ways the Guide can be mobilized into people’s lives to influence eating choices. It also presents an opportunity to consider how the Food Guide can move beyond a popular “handout” and be incorporated into evidence-based healthy eating knowledge mobilization strategies that will benefit Canadians. This could include disseminating the Guide’s nutrition messages through social media, lesson plans, videos, or self-guided workbooks that could be tailored to various age groups, demographic groups, and settings such as schools and health care facilities. These tools and approaches could help close the elusive gap between knowledge and healthy eating behaviour.

## Abbreviations

CCHS: Canadian community health survey
CFG: Canada’s food guide
